# Arts practice and disconnected youth in Australia: Impact and domains of change

**DOI:** 10.1080/17533015.2013.822397

**Published:** 2013-08-08

**Authors:** Peter Wright, Christina Davies, Brad Haseman, Barry Down, Mike White, Scott Rankin

**Affiliations:** ^a^ School of Education, Murdoch University, Murdoch, Australia; ^b^ Creative Industries Faculty, Queensland University of Technology, Brisbane, Australia; ^c^ Centre for Medical Humanities, Durham University, Durham, UK; ^d^ Big hART, Devonport, Australia

**Keywords:** impact, participatory arts, Big *h*ART, domains of change

## Abstract

*Background*: This paper describes research conducted with Big *h*ART, Australia's most awarded participatory arts company. It considers three projects, LUCKY, GOLD and NGAPARTJI NGAPARTJI across separate sites in Tasmania, Western NSW and Northern Territory, respectively, in order to understand project impact from the perspective of project participants, Arts workers, community members and funders. *Methods*: Semi-structured interviews were conducted with 29 respondents. The data were coded thematically and analysed using the constant comparative method of qualitative data analysis. *Results*: Seven broad domains of change were identified: psychosocial health; community; agency and behavioural change; the Art; economic effect; learning and identity. *Conclusions*: Experiences of participatory arts are interrelated in an ecology of practice that is iterative, relational, developmental, temporal and contextually bound. This means that questions of impact are contingent, and there is no one path that participants travel or single measure that can adequately capture the richness and diversity of experience. Consequently, it is the productive tensions between the domains of change that are important and the way they are animated through Arts practice that provides sign posts towards the impact of Big *h*ART projects.

## Introduction/Background

This paper describes phase one of a project that seeks to understand questions of “impact” in disconnected young people who take part in participatory arts by focusing on one of Australia's exemplary, and most awarded, participatory arts organisations. Big *h*ART has a long established trajectory as a provider of social impact of the Arts programmes (Wright, 2009, 2010, 2011a, 2011b; Wright & Palmer, 2007, 2009). The organisation is particularly known for its work in regional, remote and rural Australia – areas predominantly underserved by the Arts because of the challenges of distance, environment and provision (Anwar McHenry, 2009).

**Figure 1:**
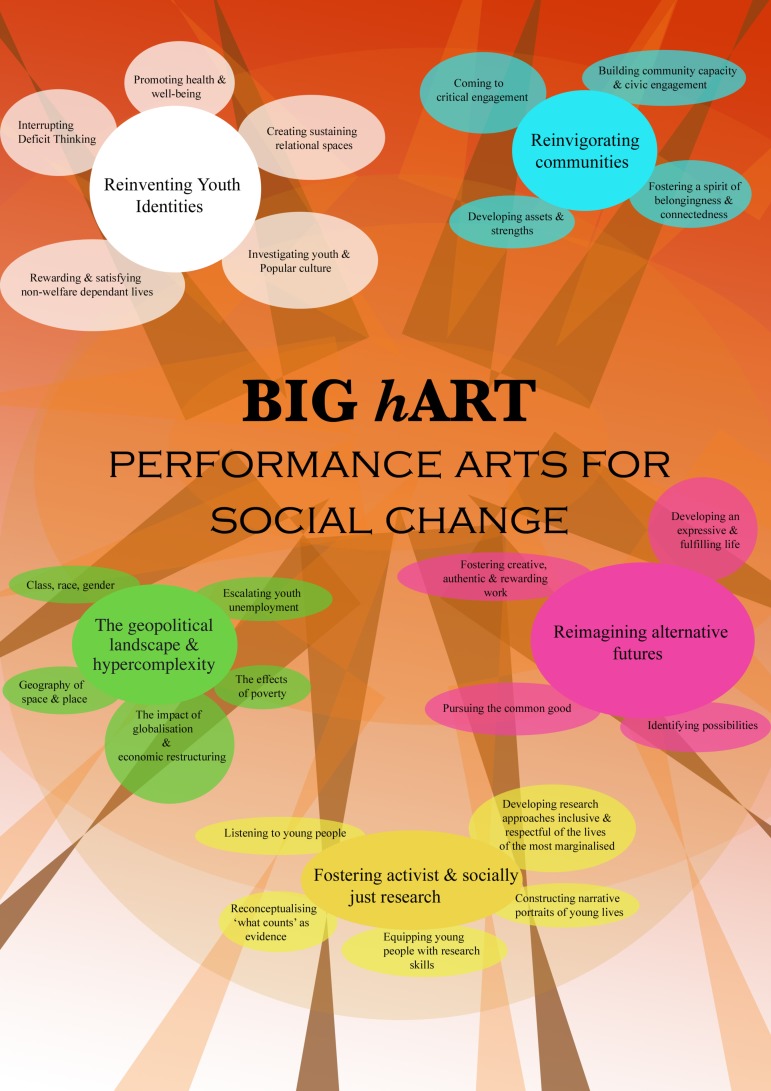
Big *h*ART Performance Arts for Social Change.

Big *h*ART works in sophisticated ways within what has been described as “socially-collaborative” (Bishop, 2007) or “socially-engaged” Art (Helguera, 2011). Key to all of this work, and consistent with the field internationally, is the way it is collaborative and informed through a “malleable dialogue” (Dix & Gregory, 2010, p. 6). Big *h*ART, with its responsive, dialogical and collaborative projects can best be understood as an “ecology of practice” (Leicester & Sharpe, 2010; Wright, 2011b), in that it is iterative, multidimensional and multi-modal. The aim of Big *h*ART projects is to “empower communities to change through the arts.” A description of Big *h*ART and its projects can be found at http://www.bighart.org/public/. Although emphasis may vary by project, each of Big *h*ART's objectives is shown in Figure 1.

Figure 1 shows that Big *h*ART exists within a particular geopolitical landscape. This intrinsically intricate context reflects globalisation, hypercomplexity – where increasing complexity represents an increasing challenge to society (Qvortrup, 2003) – and “un-knowing” in the face of uncertain futures. This particular context – where technology intersects with sociality; where the functionalities and contexts of human activity highlight trans-boundary problems with consequent concerns with food security, energy security and water security, democracy and militarisation; and where the suppression of rights and freedom exists – impacts most on the vulnerable (Smith, 2005). Big *h*ART works against these emergent realities, such as coercive power and maladaptive unlimited economic expansion, through new forms of cultural mythmaking to enable project participants to see themselves “as-if” they could be otherwise through “re-storying their lives.” As the figure reflects, this involves reinventing youth identities, re-imagining alternative futures and reinvigorating communities, while the research itself aligns with contemporary forms of activist and socially just research (Leavy, 2011).

Although the work of Big *h*ART has strong intergenerational components (MacCallum et al., 2010), projects often evolve from a focus on marginalised or disenfranchised young people. This particular demographic, sometimes referred to as the “million dollar kids” (Australian Clearinghouse for Youth Studies) because of the resources that either indirectly or directly flow to them through human service agencies, health or the justice system, has proved to be particularly resistant to what could be considered traditional forms of service provision, and continue to be considered as a deficit or drain on resources rather than appreciated as a generative resource for Australian society.

The research first describes various understandings of impact as articulated in the literature; second, checks the authenticity or resonance of these through a consideration of research sites in three different Australian states; and third, reveals how these differences can be understood. The focus of the paper was “impact” as we were interested in the short-term objectives of Big *h*ART projects rather than its long-term aim. The benefits that flow from this research include a better understanding of what impact is – with its differing attributes and dimensions – offering clearer communication and hence less mismatch between those in the work (participants), those who enable the work (Arts workers), those who see and may be touched by the work (community) and those who fund the work (including government agencies and the like), also recognising that these are never mutually exclusive and often overlap. Hence, a more sophisticated understanding of impact will benefit all those who are active in the work or are interested in supporting it through providing a clearer set of concepts, in this case “*domains of change*,” and shared understandings for consistency and clarity.

## Methods

### Recruitment and Sample

Consistent with qualitative approaches to research, sampling for proportionality was not a quality criterion. Rather, the sample was selected on the basis of interviewee characteristics and project type, in other words, what participants could reveal about the phenomena in question, but was constrained by interviewee availability and willingness to participate. Consequently, purposive sampling (Flick, 2007) was used to recruit young people who had been participants in a Big *h*ART project (*n* = 10), Arts workers who enabled the work (*n* = 7), community members who saw the work (*n* = 9) and people who worked for agencies that funded/co-funded the work (*n* = 3). With the assistance of Big *h*ART, interviewees were recruited from three separate projects across Australia, and included 13 people from Alice Springs (Central Australia – the NGAPARTJI NGAPARTJI project), 5 people from Griffith in the Murray–Darling River Basin (Eastern Australia – the GOLD project) and 11 from Tasmania (island state south-eastern Australia – the LUCKY project). Seventeen participants were female and 12 were male.

### Data Collection and Analysis

In total, 29 people participated in a semi-structured, face-to-face interview. The interview guide was developed by the research team to document the nature and type of impact the programme had on participants. We were interested in finding out how people came to be involved in the project and what they and others got from the project experience. Interview questions were open-ended and guided by the literature and programme objects. The interview guide gave participants the opportunity to retrospectively reflect on and express in detail their lived experiences. Each interview was digitally recorded with the permission of the participant. Handwritten notes were taken both during and after each interview to record emergent thoughts and ideas.

The data collection and concurrent analyses started in October 2011 and occurred over a 12-month period. Theoretical codes based on the literature were created as a starting point for the analysis. The literature search was conducted via *ProQuest*, *ERIC*, *Pubmed* and *Google Scholar* using the following keywords: “Arts,” “outcomes,” “youth” and “impact.” In total, 22 articles were reviewed and resulted in the creation of 23 deductive codes (i.e. skill development, knowledge, risk prevention, life skills, achievement, generates further opportunities, vehicle to have a voice, career pathway, income, engagement with the community, problem-solving, trust, self-esteem, cooperation, self-efficacy, critical thinking, empathy, decision-making, recognition, confidence, resilience, happiness, creativity and connection to others). The interview audio recordings were then listened to several times. These digital records rather than transcripts were chosen so that verbal factors such as tone of voice, emphasis, speed, timing and pauses – often lost when a recording is transcribed verbatim – could be included in the analysis. In addition, a narrative portrait describing the person, their experiences and incorporating interview notes was created. These narrative portraits were designed to facilitate an inter-subjective understanding of experience rather than a causal explanation and hence are closer to the intrinsically relational nature of human beings by communicating the way we describe, share and unpack the meanings in an accessible form.

A thematic analysis of the digital recordings and narrative portraits was conducted to identify concepts and overarching themes (Grbich, 2007; Miles & Huberman, 1994; Saldaña, 2009). The analysis identified both the diverse range of outcomes that participants attributed to their experiences and what was common. Following on from this stage, concepts relevant to the research question were coded into the existing theoretical nodes (based on the literature) or inductive codes were generated if new concepts emerged. Codes were combined, divided or refined as analyses progressed using the constant comparative method of qualitative data analysis (Mathison, 2005). The third stage of the analysis involved the creation of overarching themes or “domains of impact” (Dart & Davies, 2003).

### Findings

All concepts were found to cluster around one of seven overarching domains, these broadly being (1) psychosocial health – a sense of efficacy and well-being; (2) community – a sense of belonging and connectedness; (3) agency and behavioural change – the ability to act purposefully in one's life; (4) the Art – leading an expressive life; (5) economic effect – aspirations and work of value and meaning; (6) learning – strengthening capacities and dispositions; and (7) identity – cultural learning and becoming. Each domain was found to correspond and have congruence with the literature.

#### Psychosocial Health: Sense of Efficacy and Well-being

Recognising that psychosocial health and well-being are both a process – living well – and a state of being (McGregor, 2008), this domain was defined by the impact the project had on a young person's emotional health contextualised by the social-cultural nature of the project itself. Overall, the most mentioned outcome voiced by participants, Arts workers, community members and funders was the effect project involvement had on a young person's confidence, which then flowed on to self-esteem, feelings of self-worth and the influence this had on the participant's willingness to try other things. For example, as one participant observed of another: “she has changed so much, her confidence is heaps better and she is willing to get out there and try to do stuff and not let anyone tell her that she can't.” And “because she is more confident she is willing to try … she is even trying to get her driver's licence at the moment.”

This growth in confidence was also apparent to Arts workers; for example, “To engage people [in the Arts] is good … some of the people are really shy outside their own worlds so to get up on stage is great … all that stuff makes people more powerful, more confident.”

A funder described how this could be understood as “personal development” and is ultimately a generative process:

Stories of individual young people who have gone on to do other things … who have gained a huge amount of confidence from doing this kind of work and it's something that they found meaningful and purposeful so it is good for their personal development.

The Big *h*ART environment and workers were seen to empower participants, therefore changing their beliefs – and the story they could tell – about themselves and hence improving their self-efficacy. Improvements were also seen in feelings of self-image, pride, motivation and achievement as the young people were recognised for their work. One participant commented on her own improved level of self-belief: “They made us realise we could do it by ourselves.” And another highlighted how this was contingent on a non-judgemental attitude by the Arts workers themselves: “It's like they headed us in the right direction … they accepted me for who I was … they never judged me.” Another participant observation was “the fact that someone was willing to give her a go and to help her to try to do it … which is just a big confidence builder as well.” An important attribute of this dimension was the way that “recognition” of others was important.

The impact for the choir was really strong, they loved being involved and travelling and singing and they felt proud that they were a part of that … definitely their self esteem and all that really shot up … dressing up and being made to feel special and having people clap for them it was a different experience. (Arts worker)

In general, the positive affect of participants was seen to increase (i.e. happiness, enjoyment, excitement and enthusiasm), while negative affect or emotional problems were seen to decrease (i.e. anxiety, depression, unhappiness). Participants appeared to become more resilient, relaxed and calm, therefore making them more able to self-regulate their moods and emotions and more able to cope with daily stresses and adversity. One possible reason for this was that the Arts practices had meaning to the participants. As one Arts worker described, “it meant something to them and it was exciting for them to be involved.” In addition, there is a pleasure in the making. One community member suggested that “participants in the project have enjoyed themselves … it's a bit of a discovery for some people, their self-confidence can grow, belief and self-trust.” One participant also referenced the issue of how these processes are related to identity and what that meant for him: “[I found] I was becoming more myself and relaxed around everyone … I give things more of a go then I would have.”

Participants were grateful for the chance to participate in a Big *h*ART project. For many young people, participation had a positive effect on their life and gave them hope for the future. One young person talked about changes she was able to observe, and how this was also evident to others. “I can walk up the street without looking at the ground, I care about my appearance, I'm healthier, I'm studying, [others] see a lot of changes” (participant).

For some participants, however, programme gains were short-lived; the conclusion of the project made them feel “sad,” “disappointed” and abandoned, and once the project was finished they went back to life as it was. One of the Arts workers on the project also highlighted that while there were benefits for many, this was not universally the case:

She [participant] said I've got to tell you all that I've got a job. She also wanted to share about the other participants in the group. She gave me the impression that some had done really well, some had gone to university, got jobs, settled into housing that's not vulnerable. She did also mention others that have fallen by the wayside.

As one worker on a project observed of a participant beyond the project, life circumstances continued to be difficult:

One young woman I worked with from the beginning of the project was very much head down and didn't go on stage. By the end of that production, three years later, she was moving her body almost like a dance. It was amazing and yet that young person, when that project was over, you know, she just contends with her drinking problems, she's had a baby that has gone into care. (Arts worker)

What this domain reveals is the way that social structural factors beyond a project's “reach” can mediate individual psychosocial health outcomes. This means that positive impact within a project goes beyond what is often thought of as individual effort and accomplishment and reveals the importance of “relational health”(Johnson & Haigh, 2011) as one of the many pathways that influence health and well-being (Martikainen, Bartley, & Lahelma, 2002).

#### Community: Sense of Connectedness and Belonging

This domain relates to a young person's connection to and interaction with others in their community/society. While notions of community are understood differently in different contexts – physical (place), social (interaction), virtual (imagined reality), psychological (or fluid process) – the notion of “connectedness” is key (Craft, 2011). Overall, the most mentioned outcome voiced by participants, Arts workers, and community members and funders was the effect project involvement had on young people's engagement with the community. One community member noted that this had elements of reciprocity or “authentic exchange” (Fuller, 2009), and in this way was valuable in and of itself:

They are giving but they are also getting back and becoming part of the community that they have been disengaged with or has disengaged with them. Community engagement or social engagement of any kind is a worthwhile thing for people. Disengagement from society and broader experience is not good for anyone let alone young people.

More specifically, this had intergenerational benefits and reflected cultural learning (Royal Society of Arts, 2009) as “the project gave them [young people] the opportunity to reconnect and get to know their history through the older generation.”

Involvement in a Big *h*ART project changed the way participants viewed other people and how others viewed them. Changes were seen in people's attitudes and use of stereotypes. One community member highlighted the way that “[project participation] normalised certain interactions between different people … it dissolved stereotypes.” And following on, it challenged some of this person's own prejudices with surprising results:

If you don't open your eyes and you don't get out of your bubble then you never invite any new people into that bubble, then anything foreign to you will be met with either suspicion or surprise or awkwardness. I'm a lot less awkward around young people now then I was before. (Community member)

Consistent with this was a change of perceptions: “seeing the ones that carried [the project] through to the end and seeing visually a change in them, even to standing up straight … programs like this support young mums and can change perceptions” (community member). Another remarked:

A lot of Australian people are suspicious or have a “go away” attitude … but the closer you get to the coal face … well this person isn't that bad … the closer you get to the facts or the people the less fear you have.

Changes were also seen with regard to levels of trust, empathy, tolerance and respect for others. “Then the relationship between the adults and the kids are built around respect. I couldn't envision the kids hanging in there for that length of time without that respect” (community member). A funder made a similar observation:

The more we understand [young people] the less we fear them. It's hard to ignore someone if you know their story. If it ends up on television, or screenings in theatres or halls, in schools or local council chambers it makes it unavoidable and forces people to rethink what they had adopted as their opinion or attitudes towards the phenomena and [young] people involved in it.

Participants acknowledged that the programme was instrumental in increasing the quantity and quality of their social network, especially with regard to friends, peers and adult mentors. The project, in its entirety, made them feel supported, gave them the opportunity to collaborate with others and was a way of socialising, thereby helping them feel more integrated with others and less isolated. One participant highlighted it this way: “Friendships … I've made heaps of friends … people you would not have expected to become friends with.” Another revealed how some of these have been lasting:

Great for the socialising for them who didn't get out … friends, connecting, sharing our stories … having that socialising aspect and knowing there are people that care and breaking down the isolation … A lot of us still communicate heaps. We keep up to date with what each other is doing just through what you post as your [Facebook] status … there is also a chat option to see what's going on … we need to do a reunion soon. (Participant)

And it is these connections, which for many were the most significant outcome of participation. As one young person explained:

The most significant change through the project is those connections with others … for those who don't reach out to be involved in something where they have that chance to reach out and know that there is people who are going to listen and not judge what they are saying. (Participant)

The project was also a platform for social justice in the way it gave young people the opportunity to belong to something bigger then themselves. In addition, the project gave young people a voice and the opportunity to reflect on their lives and role in society. One community member highlighted, “[The Arts] gives young people a voice. Makes them more visible to society. It's a vehicle to explore their personal values, judgements, risk taking behaviour consequences.” An Arts worker on a project described how she saw this happen:

If they stayed at home they would be sitting around either participating in or exposed to really dysfunctional and destructive behaviour like drinking and violence. If we got young people like that to come in, to use the computers to make something like posters, digital stories, movies, it gives them an opportunity to reflect or just engage. Sometimes some of those young people just liked coming in and looking at photos of themselves. They talk about how everyone listened to them. They had such obvious pride in looking in those photos. For us that was a success that someone would come in, feel comfortable enough to come in and sit and process that experience they had.

What this domain reveals is how both quality and quantity of social connections are key to understanding benefits of participatory arts through enhanced opportunities for, and innovative forms of, connectivity.

#### Agency and Behavioural Change

This domain relates to a young person's sense of agency and being able to act positively upon the world. In general, projects gave young people the opportunity to learn or strengthen health-seeking (and affirming) behaviours and reduced disruptive, violent or risky behaviours. This included, for example, their participation in unlawful activities and use of drugs and alcohol. For example, one young person recounted: “I was in the wrong circle of friends. I branched out. I knew there was more. The programs helped me … I was taking drugs and living an out there lifestyle … it [the project] settled me down” (participant). An Arts worker on the project described how a creative “option” engaged these young people, providing a different life-choice:

[We] engage minds creatively at a point where they [young people] were dropping out of school, where they were pretty disinterested and angry about things and starting to slip into crime. They just needed someone to keep them out of jail and I think that is what we did, they were heading down the juvenile justice route.

Funders, in the same way, saw that positive health behaviours were critical. One recounted: “There are big issues here regarding avoidable chronic disease, youth suicide, adult literacy, the binge drinking culture amongst young people.” What was evident across this domain was the way creativity manifests itself as important ability, or capacity, to act on the world in health-increasing or health-sustaining ways.

#### The Art: Leading an Expressive Life

This domain relates to the impact creating Art has on participants, and it is the core domain around which the others sit. Involvement in a Big *h*ART project gave disconnected youth the chance to interact with the Arts community and artist mentors, and access creativity past and present. Project participation gave young people the opportunity to be creative and act as artists; this creative act being constructive, to participate in something that took them beyond themselves, allowed them to escape what were often difficult and challenging conditions, and to produce work that was valued by society. Big *h*ART projects provided participants with Art skills and taught good Arts practice. The creation of Art, visual, electronic, literature or performance-based, gave participants the opportunity for self-expression, aesthetic satisfaction and the desire and freedom to be creative; in short, to lead “expressive lives” (Jones, 2009) that reflect meaning with creativity as a core value. These abilities, strengthened through Arts practices, make a virtue of flexibility, fluidity, change and responsiveness, all attributes valued in the twenty-first century (www.p21.org).

While, simplistically, this work could be seen as “diversionary” – “It gave them [young people] something to do,” it was much more than this and reveals the power of “making,” “Making something makes you feel good” (participant), and the importance of expression: “Through the magazine we did writing and expressed our feeling through the writing” (participant). What this highlights is the broad skills and developmental possibilities of projects that attracted and held the interest of young people – from diversion (Polk, Adler, Muller, & Rechtman, 2003) towards rapt attention and captivation, or transcendent experience with positive outcomes. One Arts worker described her experiences this way:

The software that you use [for creative production] contributes to that sense of agency for young people who don't have English Literacy. Even doing things like a little film they could choose the title, choose the colour of the font. For people who haven't got the experience of being offered a lot of choice because they feel that they can't read or write, they could easily, without extensive literacy, make choices about how they wanted to represent themselves.

However, a negative of the way Big *h*ART projects were presented was that some interviewees thought that the Art was often presented in a “western format” and judged by western values, this being particularly true when working in indigenous communities who do not always have English as their first language, or a western sense of aesthetics. One Arts worker highlighted the challenges this entailed:

It was interesting because the project was talked about as a cross-cultural project. What wasn't really acknowledged was that we were rolling out a very western cultural tradition which was theatre and all of its production, timetable and values and it was really just to fit in. There was no space for anything else. These kids never have anything like that in their lives. They just roll with the day as it goes. This schedule was relentless. It was really hard. People were exhausted; that level of concentration for hours in English.

#### Economic Effect: Aspirations and Work of Value and Meaning

This domain relates to a young person's resources, money and career prospects. For some participants, participation in a Big *h*ART project had a positive impact on their career prospects and career goals. One young person highlighted this in their aspirations: “Before I went to Big *h*ART I didn't have any future goals. Now I'd like to own a clothing company, create a clothing line” (participant). For others, project participation improved their employment prospects, led to employment opportunities and gave them the confidence to try other courses/projects. As one young person noted: “it led to my job and where I am now,” and another noted more specifically:

[it] let the participants know that there is things out there for them and that they can have opportunities. We did our tourism certificate through that so everyone got a certificate from that so we can be the guides on tour busses and things like that with that qualification. So it gave them a qualification and to know that they can do something. (Participant)

These benefits were also noticed by those in the community. For example, “they go off to find jobs or go back to do re-education or get involved in other community projects” (community member). These aspirations are also influenced by what is valued by the society in which they live; for example, cultural experiences and expectations influenced project participants differently across research sites in Tasmania, Alice Springs and western NSW.

#### Learning: Strengthening Capacities, Dispositions and Skills

In addition to Art-specific skills, participants indicated that project participation was a form of learning that improved their general knowledge, skills and capabilities. One young person, for example, “learnt a few cooking skills” (participant), while others “picked up a range of skills … literacy skills, social skills, [and] the understanding of mutual obligation” (Arts worker). As one participant observed: “My public speaking improved cause I always got dobbed in to do the speaking.” Gains were observed in young people's communication skills, linguistic ability and literacy skills. Improvements were also seen in the participants' leadership skills, ability to process information, solve problems and make decisions. In addition, participants showed improvements in their motivation, attitudes and levels of concentration. One young person observed of another:

She actually does a full time course at TAFE now to do aged care. She is on her second year of that and this is someone who has never done anything with her life before. It [the Arts project] got her out there doing something. (Participant)

That participants could observe this in a personally reflective way, and also in other project participants, was particularly revealing and highlighted increasing levels of self-awareness.

What I've been given from the project is personal things like communication skills … I didn't communicate properly, I had trouble working with people and one of the major things I got from Big *h*ART was working with people not just on a physical level but at a creative level. (Participant)

#### Identity: Cultural Learning and Becoming

This domain relates to a person's expression of and sense of self, either as an individual or as part of a group, as well as understanding their own or others' beliefs, values, language and customs. Overall, Big *h*ART projects were seen as a platform to explore and express an individual's cultural identity as well as a way of experiencing other cultures; the performance scholar Taylor (2003, p. xviii) highlights the power of *performing* collective cultural memory as a way of “reorientating social memory and cultural identity.” One young person, for example, talked about his own developing sense of self:

It changes the way you understand who you are. People didn't forget about the show straight away. They thought about it and talked about it after it was on. They really took it on inside, they had feelings about it and shared that with each other. (Participant)

This notion of “knowing who you are” was particularly profound for indigenous participants. As an Arts worker on one indigenous project noted:

In 100 years their descendants can watch them talking. Speaking in their own language, promoting their own culture and promoting their own work. The results are extraordinary.

This domain was particularly significant in the way that it exemplified cultural learning – the projects in one sense being seen as *cultural interventions*. For example, one community member explained: “The project gave them [young people] the opportunity to reconnect and get to know their history through the older generation.”

## Discussion

This research reveals a comprehensive picture of what constitutes impact across three Big *h*ART projects and a mix of stakeholders and so reveals both a conceptual terrain and influence of practice. It is also the case that while each research site was embedded in a particular cultural context, and in this way defined by “place” and mediated by local cultural practices and perspectives, they were also linked through understandings of disadvantage, including personal, social, material and cultural (Price-Robertson, 2011). Key to this understanding are the links, now well established, between sociality – both in quality and quantity – disadvantage, health outcomes and access to structural and cultural opportunities (Umberson & Montez, 2010; Vinson, 2009).

The research highlights the way that Arts practice – through creation and (re)creation – is connecting of ideas, biographies and materials, and how the bonds that are established can mitigate against some of societies' inequality structures. Re-storying one's life or biography, while not ameliorating material inequality, can enrich both the quantity and quality of one's relationship with others, with associated human health benefits and contribution towards a more hopeful future (Elstad, 1998; Wright, 2012).

Second, is the way that the domains of change are iteratively concerned with the processes of meaning making. One of the key ways that meaning is promoted and communicated is through the Arts and the social structures and experiences that surround them. It is these two (of three) features – meaning and social engagement – that research in positive psychology and happiness studies highlights as contributing to what makes a “good” life (Seligman, 2002). It is also interesting to note that the third key feature, positive emotion, also flows out of these experiences and comes from the joy of making one's mark on the world, and the recognition of others – all significant features of Big *h*ART's work.

Linking with notions of sociality is the way that the domains reveal individual identity as formed by one's heritage and relational ways of knowing. For example, cultural learning is always implicit in the Arts, and it can be understood in both the way that it enables access to one's own heritage, but it also provides the capacity to contribute to it highlighting the way that culture and identity are dynamic and iterative. Disconnected young people are often literally and socio-culturally “outsiders” and we consistently observed, across each research site, a negotiation through participation of what had been a devalued identity moving towards a greater sense of value and coherence that Antonovsky (1990, 1998) describes as helping explain movement towards the health end of the health/illness continuum.

It is this notion of “movement” – and ultimately reciprocity – that reflects a sense of agency and the ability to act on the world through creation and (re)creation, recognisable across human history as the desire to be recognised. What the research reveals is the way that a stronger sense of self, agency and the skills and knowledge developed are enabled through Arts practice. Arts practices, with emphases on both play and expression – that is *human experience –* are the key enablers of agency both through what has been described as “flow” or “optimal experiences” (Csikszentmihalyi, 1990) and the power to “do and be more” through action, engagement and intersubjective and intrasubjective understanding (Wright, 2011a).

Key to this understanding of participatory arts is contemporary understandings on the role of Art that sees meaning as being determined by context, historiography and relative values rather than an aesthetic that is immutable and consistent over time. The question of “value” is central to this debate as value in our general lexicon is usually reduced to monetary value (Self, 1975) and underlies many questions driving measurement and outcomes, hence “impact,” in the field. However, as Sharpe (2010) and Leicester and Sharpe (2010) describe, to reduce all value to monetary value is not real. There is overwhelming evidence that what people care deeply about has little to do with money at all, but rather common human needs for social, psychological and physical well-being. It is these values that influence “most, if not all, human behaviour” (Schwartz, 2006, p. 17), with many of these values serving both individual and collective needs (Schwartz, 1990). Consequently, the broader suite of domains of change described here point to the multi-valent benefits that potentially accrue from the immersive experiences characteristic of participatory arts.

It is through building creative platforms for social mobilisation that Big hART can deliver such a high level of outcomes in the lives of participants, and that this can occur across such a wide array of domains is the result of an imaginative act made manifest. These diffuse outcomes are particularly powerful in the way that they add “value” to addressing government policies and provide creative solutions to intractable social issues. It is also important to understand that these “social” dimensions are not set in a binary opposition to the Arts outcomes that are intrinsic to the work. In the same way that fabric is made up of the warp and weft of threads combined – the sum being greater than the parts – so too is the production of Art not discrete or away from how participants engage in the work but central to it. Understanding processes within the work and potentialities as outcomes means that there is not a single “measure” that can adequately capture impact, nor is there a single pathway by which participants travel.

This means that impact cannot be accounted for in a single way, nor are participant's experience of participatory arts unified. For example, one person may return to education or gain employment for the first time, another may improve both the quality and quantity of their social networks with benefits accruing over time. Mastering a particular skill set or feeling like one has a voice can have generative consequences. Simply developing a significant relationship with one other person can begin a process of reframing one's own identity through meaningful interactions.

Key to understanding impact is the way that experiences of participatory arts are interrelated in an ecology of practice that is iterative, relational, developmental, temporal and contextually bound. Consequently, single measures at one point in time do not adequately reveal the way that benefits flow from participation and each of the seven domains of change need to be considered when considering the influence of a participatory arts project. It is also the case that because “place” matters, different vantage points and different contexts will foreground some domains and not others. While, formally, each of these domains of change could be conceptualised of as an independent entity, what is clear is that it is the interaction between them – the relational component and the productive tensions between them, in short the “fuzzy edges” – that is important. And it is the Arts practices of creating and (re)creating that enable this interaction to happen in powerful ways.

This research, while located across three different Australian research sites – each culturally diverse – is also delimited by considering one company and also one particular group of project participants, principally in this case, young people. There are broader cross-cultural issues, and each situated project has a temporal component that will define the work it does. This means that as cultural communities profoundly shape the perspectives of those within them, so it is the case that different domains will be foregrounded or backgrounded across differing contexts. Speaking back and resistance against oppression, for example, can be equally as important for positive youth development as are developing practices for coping and adapting.

In short, effective participatory arts projects, of which Big hART is an exemplary provider, influences change across seven broad domains. These seven domains of change provide a coherent and interpretable framework within which it is possible to consider impact, and the increasing ability of participants to act autonomously and in life-affirming and life-enhancing ways. Consequently, a consideration of these domains helps us better understand where we might look for markers of impact and what a meaningful life well-lived might actually mean.

Finally, Big hART engages in facilitating a creative discourse around their work and an exploration of a critical aesthetic pedagogy that generates exemplary cultural experiences. While the benefits that flow from being creative cannot be prescribed from a doctor's notepad, knowing what some of these benefits might be and where we might look for them enables us to be better informed as to how we might describe, understand and provide opportunities for these in the future.
